# Comparative mortality outcomes in metabolic dysfunction-associated steatotic liver disease and nonalcoholic fatty liver disease subtypes in the United States

**DOI:** 10.1371/journal.pone.0335230

**Published:** 2025-10-31

**Authors:** Pengwei Zhang, Sijia Yang, Rong Hu, Tianfang Peng, Peipei Yu, Yijun Zeng, Chunhong Ye, Panpan Wang, Xianhui Dong, Zhiying Che

**Affiliations:** 1 Hubei University of Chinese Medicine, Hubei, China; 2 Haining Traditional Chinese Medicine Hospital, Zhejiang, China; 3 The Affiliated Hospital of Hangzhou Normal University, Hangzhou Normal University, Hangzhou, China; 4 Pingxiang Health Vocational College, Jiangxi, China; 5 Hangzhou Normal University, Hangzhou, China; 6 Traditional Chinese Medicine (Zhong Jing) School, Henan University of Chinese Medicine, Zhengzhou, China; Tehran University of Medical Sciences, IRAN, ISLAMIC REPUBLIC OF

## Abstract

**Background:**

In 2023, experts from the European and American regions proposed the concepts of steatotic liver disease (SLD) and metabolic dysfunction-associated steatotic liver disease (MASLD). MASLD was proposed as a replacement for nonalcoholic fatty liver disease (NAFLD). We compared the long-term outcomes of patients with MASLD, NAFLD, and various subtypes of SLD.

**Methods:**

We conducted a retrospective study using the NHANESIII database. Cox proportional hazards models were used to estimate hazard ratios (HRs) and 95% confidence intervals (CIs) for all-cause mortality and cause-specific mortality among patients with subtypes of SLD, MASLD, and NAFLD.

**Results:**

During a follow-up period of 31 years (median 25 years), the adjusted risks of all-cause death for patients with MASLD was 1.19 (95% CI 1.06–1.34; *P* = 0.006) *vs*. the non-SLD group. There was a moderate level of consistency between MASLD and NAFLD (Cohen’s kappa coefficient of 0.62545). Advanced fibrosis was the most serious risk factor for all-cause mortality in MASLD, and high C-reactive protein concentration was the most serious risk factor for all-cause mortality in NAFLD, followed by type 2 diabetes.

**Conclusions:**

MASLD is associated with a higher risk of all-cause mortality, and this association is independent of patients’ demographic or metabolic characteristics, despite a relatively small hazard ratio. Our research findings further support that MASLD is a pathological disease related to liver disease itself. Therefore, redefining NAFLD as MASLD may help improve our understanding of predictive factors that increase the risk of death.

## Introduction

Nonalcoholic fatty liver disease (NAFLD) is the most common chronic liver disease, and currently affects 38% of the global population [1]. NAFLD exerts negative effects on both the liver and extrahepatic systems and is an independent risk factor for all-cause mortality and adverse cardiovascular outcomes [2–5]. The name NAFLD includes ‘non-alcoholic’ and ‘fatty’, which are potentially stigmatizing terms and underemphasize the significance of metabolic risk factors in the progression of the disease [6–8]. Therefore, Rinella *et al* [9] suggested the renaming of NAFLD as “metabolic dysfunction-associated steatotic liver disease” (MASLD) in 2023 and proposed the use of “steatotic liver disease” (SLD) as a generic term to encompass the various causes of steatosis.

The introduction of this new vocabulary and the associated diagnostic criteria were timely and important, and these new definitions have been warmly embraced in both Europe and the US. The MASLD criteria, centered on cardiometabolic risk factors, promise a more precise identification of individuals whose fatty liver disease is driven by metabolic dysfunction, potentially refining patient diagnosis beyond the simple exclusion of significant alcohol use [10]. This enhanced phenotyping may lead to improved risk stratification by better capturing the intrinsic metabolic risk associated with adverse hepatic and extrahepatic outcomes [11]. Therefore, the definition of MASLD will shape SLD management strategies, promoting comprehensive approaches that target both underlying metabolic drivers and associated liver dysfunction.

It remains unclear how the clinical characteristics, prognosis, and mortality risk stratification differ across SLD subtypes, especially in light of the shift from NAFLD to the positively defined MASLD criteria. Therefore, we used the National Health and Nutrition Survey 1988–1994 (NHANES III) database to investigate the clinical characteristics and mortality of patients with four distinct subtypes of SLD, and compared the all-cause and etiology-specific mortality rates of patients who fulfilled the definitions of MASLD and NAFLD, in order to better understand SLD and MASLD, ultimately aiming to provide evidence that may inform more personalized risk stratification and management strategies for patients with hepatic steatosis.

## Materials and methods

### Data source and study sample

**Fig 1:**
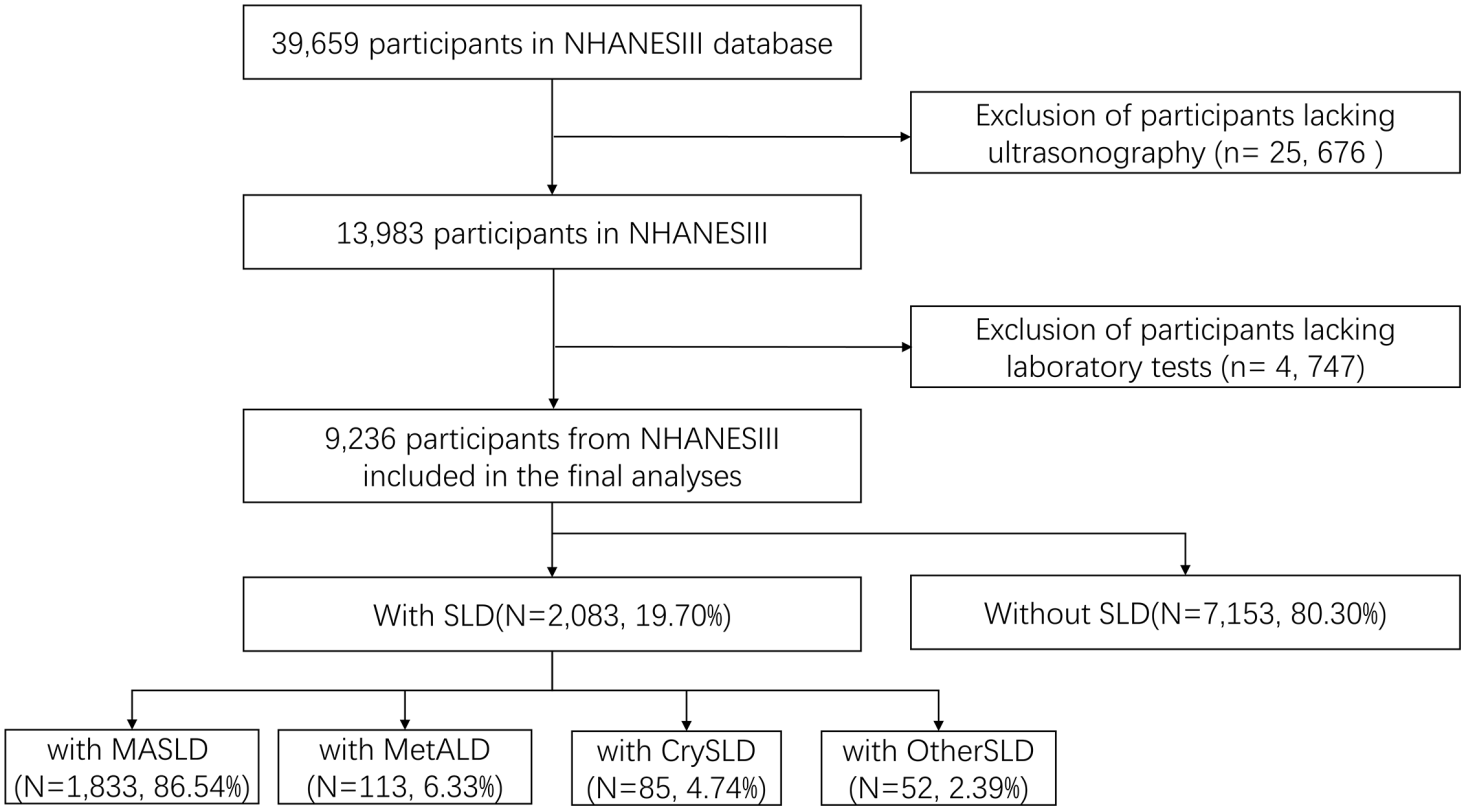
Flow-chart for the subjects’ selection. SLD, steatotic liver disease; MASLD, metabolic dysfunction-associated steatotic liver disease; MetALD, Metabolic and alcohol-associated liver disease; CrySLD, cryptogenic ALD; OtherSLD, Other specific aetiology SLD.

The raw data were sourced from the publicly accessible NHANES III database (1988–1994), which was conducted by the US CDC’s National Center for Health Statistics (NCHS) using standardized protocols administered by trained medical personnel. These protocols encompassed home interviews, physical examinations in mobile centers (MECs), and laboratory specimen analysis. Of the 13,983 adults aged 20–79 years who underwent liver ultrasound examinations, we excluded participants with ungradable ultrasonographic images (n = 127), missing mortality data (n = 10), missing laboratory measures (including blood glucose, insulin, glycosylated hemoglobin, alanine aminotransferase, creatinine, triglycerides, total cholesterol, high-density lipoprotein cholesterol, and C-reactive protein) or physical examination measurements (including body mass index, waist circumference, waist-to-hip ratio, systolic blood pressure, and diastolic blood pressure) (n = 3948), and those with missing data on alcohol consumption or hepatitis serology (n = 662), resulting in a final analytical sample of 9,236 participants (Fig 1). The NHANEIII survey protocol was approved by the Institutional Review Board (IRB) with written consent from participants. The data are available at https://www.cdc.gov/ nchs/nhanes/index.htm.

### Variables and outcomes

Data regarding the age, sex, ethnicity, smoking and alcohol consumption statuses, history of diabetes and hypertension, and history of medication of the participants were collected from the NHANESIII household adult file data. Daily alcohol intake was calculated according to participants’ “days of drinking in the past year” and “how much alcohol they drank in a day on average”(1 drink = 14 g ethanol). Smoking was defined as having smoked at least 100 cigarettes in life and still smoking.

The following variables were collected from the examination and laboratory data: body mass index (BMI), waist circumference, waist-to-hip ratio (WHR), systolic blood pressure (SBP), diastolic blood pressure (DBP), high-sensitivity C-reactive protein (CRP), glycosylated hemoglobin (HbA1c), blood glucose (BG), insulin (INS), aspartate aminotransferase (AST), alanine transaminase (ALT), total cholesterol (TC), triglyceride (TG),albumin (ALB), total bilirubin (TBil), high-density lipoprotein-cholesterol (HDL-C), blood urea nitrogen (BUN), serum creatinine (Scr), C-peptide (CP), uric acid (UA), alkaline phosphatase (ALP).

Simultaneously, we obtained data regarding death as of December 31, 2019, that corresponded to the NHANESIII data; these data included follow-up time, survival status, and underlying leading causes of death. Among the causes of death, we focused on CVDs and malignant tumors.

### Diagnostic criteria and definitions

SLD was diagnosed using the recode variable relating to the results of an ultrasonographic examination. Hepatic ultrasonography was performed on NHANES III (1988–1994) participants using the Toshiba Sonolayer SSA-90A system (Toshiba America Medical Systems, Tustin, CA). The results were categorized as yes or no for hepatic steatosis. Those with hepatic steatosis (yes) were classified as having steatotic liver disease (SLD) [12]. The use of the recode helps avoid the potential overlap between mild and moderate hepatic steatosis. Patients with SLD who met the cardiometabolic risk factor (CMRF) criteria and consumed ≤30 g/d alcohol (men) or ≤20 g/d (women) were classified as having MASLD;

CMRF were defined as the followings: a) BMI ≥ 25 kg/m2 [23 kg/m2for Asia] or waist circumference ≥ 94/80 cm for men and women; b) fasting serum glucose of ≥ 100 mg/dL or HbA1c ≥ 5.7%) or type 2 diabetes or treatment for type 2 diabetes; c) blood pressure ≥ 130/85 mmHg or specific antihypertensive drug treatment; d) plasma triglycerides ≥ 150 mg/dL or lipid lowering treatment; and e) plasma HDL-cholesterol <40 mg/dL for men and <50 mg/dL for women or lipid lowering treatment [10].

Those with SLD who met the CMRF criteria but consumed 30–60 g/d alcohol (men) or 20–50 g/d (women) were classified as having Metabolic and alcohol-associated liver disease (MetALD); those with SLD who met the criteria for ALD were classified as other specific aetiology SLD (OtherSLD), with or without CMRF; and the remaining patients with no identifiable cause for their disease were classified as having cryptogenic ALD (CrySLD) [9].

NAFLD was defined using the presence of hepatic steatosis on hepatic ultrasonography and the lack of heavy alcohol consumption (alcohol consumption < 20 g/day for males and 10 g/day for females) or viral hepatitis (serological testing shows viral hepatitis C and viral hepatitis B) [13].

We created four groups using a combination of the MASLD and NAFLD definitions: (1) a group comprising individuals who met the definitions of both MASLD and NAFLD (MASLD + /NAFLD+); (2) a group comprising those with MASLD but not NAFLD (MASLD + /NAFLD−); (3) a group comprising those with NAFLD but not MASLD (MASLD − /NAFLD+); and (4) a group comprising those with neither MASLD nor NAFLD (MASLD − /NAFLD−).

Hypertension was defined having the history of hypertension, the use of antihypertensive medication, SBP ≥ 130 mmHg, or DBP ≥ 80 mmHg [14].

Type 2 diabetes mellitus (T2DM) was defined having the history of T2DM, using a HbA1c ≥ 6.5%, or the use of hypoglycemic medication. Fasting blood glucose, 2-hour postprandial blood glucose were not available for data collection.

The assessment of liver fibrosis utilizes the FIB-4 score, the recommended non-invasive method in guidelines [10]. The Fibrosis-4(FIB-4) score was calculated as follows: age [years] ∈ AST [U/L]/ ((platelet count [10^9^/L]) ∈ (ALT [U/L]^1/2^) [15]. FIB-4 ≥ 2.67 defined patients with risk for advanced liver fibrosis [16].

### Statistical analyses

The NHANESIII used a complex, multi-stage probability sampling design to select participants that were representative of non-institutionalized US citizens. Therefore, sample weighting was used in all the statistical analyses performed in the present study. Specifically, the sample weighting utilized the MEC examination sample weight (WTPFEX6). Continuous variables are expressed as mean and standard error, and categorical variables are expressed as percentages. One-way analysis of variance and the Kruskal-Wallis test were used to compare the data for the various SLD subtypes and participants with the various combinations of MASLD and NAFLD definitions. Subsequently, for variables that showed significant differences in the initial analyses, regression analysis was used to compare data among the MASLD, MetALD, OtherSLD, CrySLD, and No-SLD groups; and among the MASLD + /NAFLD + , MASLD + /NAFLD − , MASLD − /NAFLD + , MASLD − /NAFLD − , and No-SLD groups.

We used Cox regression models to calculate hazard ratios (HRs) and 95% confidence intervals (CIs) for all-cause, CVD-related, and cancer-related mortality rates for the various SLD subtypes and patients fulfilling the various combinations of MASLD and NAFLD definitions. Covariates significantly associated with survival time were identified through statistical testing and validated for biological and medical plausibility to ensure meaningful interpretation of survival outcomes. We used three Cox regression models for the various SLD subtypes: Model 1 was adjusted for age, sex, and ethnicity; Model 2 was adjusted for age, sex, ethnicity, alcohol consumption, smoking, and hepatitis; and Model 3 was adjusted for the variables in Model 2, plus diabetes mellitus, hypertension, high TG concentration, low HDL-C concentration, high CRP concentration, obesity, and TBil, ALB, AST, and ALT. In addition, we further adjusted the physical activity and poverty income ratio. For the Cox regression analyses of the patients fulfilling the various combinations of MASLD and NAFLD definitions, Model 1 was adjusted for age, sex, and ethnicity; Model 2 was adjusted for age, sex, ethnicity, and smoking; and Model 3 was adjusted for the variables in Model 2, plus diabetes, hypertension, obesity, TBil, ALB, AST, and ALT. In addition, we further adjusted the physical activity and poverty income ratio. All Cox regression models met the proportional hazards assumption. Data were analyzed using R v.4.1.2.0 (R Foundation for Statistical Computing, Vienna, Austria), incorporating survey weights from NHANES III’s complex survey design through the utilization of the survey package. We used the false-discovery rate (FDR) correction to adjust for multiple tests in the regression models. All P-values were two-tailed, and FDR-corrected P < 0.05 was used to define statistical significance.

### Ethics statement and approval and consent to participate

The Institutional Review Board of the Centers for Disease Control and Prevention approved the NHANSIII investigation, and written informed consent was obtained from all the participants.

## Results

### Study population

**Fig 2:**
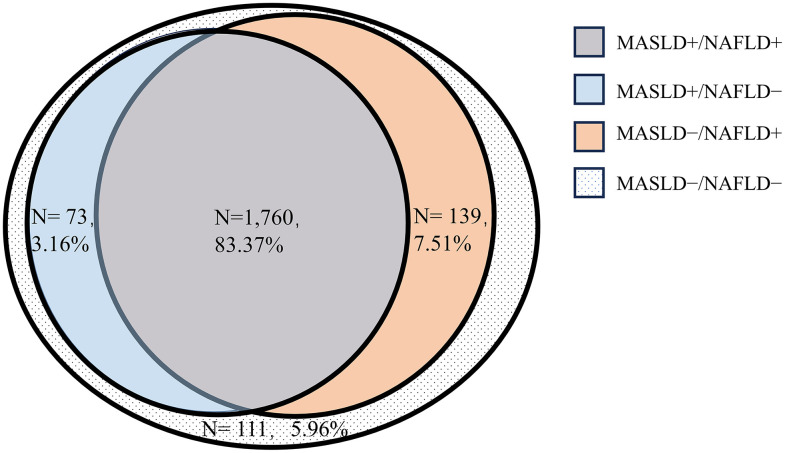
Relative proportions of MASLD and NAFLD among individuals with SLD. MASLD + /NAFLD + , a group comprising individuals who met the definitions of both MASLD and NAFLD; MASLD + /NAFLD − , a group comprising those with MASLD but not NAFLD; MASLD − /NAFLD + , a group comprising those with NAFLD but not MASLD; MASLD − /NAFLD − , a group comprising those with neither MASLD nor NAFLD.

We performed a cross-sectional analysis of data from 9,236 participants in the NHANESIII (1988–1994) database and the death database, of which 2,083 had ultrasonographically defined SLD. Those with SLD comprised 1,833 participants with MASLD, 113 with MetALD, 52 with OtherSLD, and 85 with CrySLD (Fig 1). According to the definitions of MASLD and NAFLD, there were 1,760 participants in the MASLD + /NAFLD+ group, 73 in the MASLD + /NAFLD− group, 139 in the MASLD − /NAFLD+ group, and 111 in the MASLD − /NAFLD− group (Fig 2).

### Distribution and characterization of the various subtypes of SLD

The overall weighted prevalence of SLD was 19.70%. Among participants with SLD, the prevalence of MASLD was 86.54%, that of MetALD was 6.33%, that of CrySLD was 4.74%, and thact of OtherSLD was 2.39%, as shown in Fig 1. The demographic characteristics of the participants without SLD and with the various SLD subtypes are presented in Table 1. Compared with individuals without SLD, those with MASLD, MetALD, and OtherSLD were older, were more likely to be male, were more likely to have diabetes and hypertension, had a shorter survival time, and had higher mortality. In addition, we compared the body measurement data and blood biochemical indicators between MASLD participants and participants with other subtypes of SLD (S1 Table). Compared to individuals without SLD, those with MASLD, MetALD, and OtherSLD exhibit higher levels of UA, AST, ALT, C-peptide, SBP, DBP, as well as increased waist circumference and waist-to-hip ratio.

**Table 1:** Comparison of baseline characteristics of subjects with different subtypes of SLD.

Variable	Total	No-SLD	CrySLD	MASLD	MetALD	OtherSLD	P value	CrySLD VS No-SLD	MASLD VS No-SLD	MetALD VS No-SLD	OtherSLD VS No-SLD
**Age, years**	43.42(0.39)	42.40(0.40)	39.46(1.92)	47.87(0.59)	49.47(1.50)	49.08(2.13)	**< 0.0001**	0.13983	**< 0.001**	**< 0.001**	**0.00254**
**Race, %**							0.62				
White	6273(85.44)	4715(84.87)	52(84.36)	1377(87.57)	90(92.50)	39(90.04)					
Black	2666(10.60)	2217(11.08)	28(11.42)	389(8.68)	19(5.46)	13(9.96)					
Other	297(3.95)	221(4.05)	5(4.22)	67(3.74)	4(2.03)	0(0.00)					
**Sex, %**							**< 0.0001**	0.17346	**0.00263**	**0.018227**	**< 0.001**
Female	4401(51.45)	3313(47.10)	34(32.66)	930(53.81)	80(68.00)	44(85.79)					
Male	4835(48.55)	3840(52.90)	51(67.34)	903(46.19)	33(32.00)	8(14.21)					
**T2DM, %**							**< 0.0001**	**< 0.001**	**< 0.001**	**< 0.001**	**0.001935**
Normal	8235(92.92)	6609(95.26)	85(100.00)	1409(82.60)	89(81.85)	43(83.26)					
T2DM	1001(7.08)	544(4.74)	0(0.00)	424(17.40)	24(18.15)	9(16.74)					
**Hypertension, %**							**< 0.0001**	**< 0.001**	**< 0.001**	**< 0.001**	**0.001**
Normal	5400(63.18)	4525(68.23)	85(100.00)	1332(73.38)	76(60.20)	30(58.90)					
Hypertension	3836(36.82)	2628(31.77)	0(0.00)	501(26.62)	37(39.80)	22(41.10)					
**Obesity, %**							**< 0.0108**	**< 0.001**	**< 0.001**	**< 0.001**	0.5379
Normal	5559(54.15)	3859(47.59)	0(0.00)	1577(85.80)	95(84.12)	28(53.77)					
Obesity	3677(45.85)	3294(52.41)	85(100.00)	256(14.20)	18(15.88)	24(46.23)					
**Smoking, %**							**< 0.001**	0.714	**< 0.001**	0.405	0.575
Normal	6659(69.66)	5064(68.16)	56(71.55)	1445(77.34)	68(62.12)	26(63.20)					
Smoking	2577(30.34)	2089(31.84)	29(28.45)	388(22.66)	45(37.88)	26(36.80)					
**Hepatitis, %**							0.78				
Normal	8965(97.47)	6929(97.43)	85(100.00)	1793(97.59)	110(96.26)	48(96.41)					
Hepatitis	271(2.54)	224(2.57)	0(0.00)	40(2.41)	3(3.74)	4(3.59)					
**FIB-4 score**	0.94(0.02)	0.91(0.02)	0.96(0.09)	1.01(0.03)	1.27(0.15)	1.59(0.20)	**< 0.0001**	0.59531	**0.00861**	**0.01878**	**0.00145**
**FIB-4 category, %**							**< 0.0001**	0.77896	0.12936	**0.000912**	**< 0.0001**
FIB-4 ≥ 2.67	178(1.39)	126(1.14)	1(0.86)	35(1.75)	6(7.21)	10(16.90)					
FIB-4 < 2.67	9058(98.61)	7027(98.86)	84(99.14)	1798(98.25)	107(92.79)	42(83.10)					
**Time, month**	300.50(2.36)	305.57(2.60)	324.26(3.21)	281.05(3.12)	244.60(10.64)	241.16(21.32)	**< 0.0001**	**< 0.001**	**< 0.001**	**< 0.001**	**0.00341**
**Status, %**							**< 0.0001**	**0.0101**	**< 0.001**	**< 0.001**	**0.0013**
Alive	6007(70.59)	4876(73.65)	75(91.02)	981(57.58)	53(46.37)	22(43.11)					
Death	3229(29.41)	2277(26.35)	10(8.98)	852(42.42)	60(53.63)	30(56.89)					

Abbreviations: SLD = steatotic liver disease, MASLD = metabolic dysfunctional associated fatty liver disease, OtherSLD = other specific aetiology SLD, CrySLD = cryptogenic ALD, T2DM = Type 2 diabetes mellitus, FIB-4 = Fibrosis-4.

### All-cause mortality of participants with the various subtypes of SLD

During a follow-up period of up to 31 years (median 25 years), among subjects with SLD and subjects with No-SLD, there were 952 and 2277 deaths from all causes. Among individuals with SLD, there were 852, 60, 30, and 10 deaths of any cause; as well as 240, 16, 7, and 1 cardiovascular-related deaths; and 192, 14, 7, and 4 cancer-related deaths among the participants with MASLD, MetALD, OtherSLD, and CrySLD, respectively. As shown in Table 2, the all-cause mortality of the MASLD group was higher than that of the SLD group (adjusted HR, 1.19; 95% CI 11.06–1.34; *P* = 0.006), the all-cause mortality of the MetALD group was 2.21 times higher than that of the SLD group (adjusted HR, 2.21; 95% CI 1.48–3.29; *P* = 0.004), and the all-cause mortality of the OtherSLD group was still higher (adjusted HR 2.40; 95% CI 1.43–4.04; *P* = 0.013). These associations remained statistically significant after adjustment for physical activity levels and poverty-income ratio (S2 Table). Although the unadjusted risk of death of the CrySLD group was lower than that of the No-SLD group, the all-cause mortality of the CrySLD group did not differ from that of the No-SLD group after adjustment for variables including demographic parameters and metabolic risk factors.

**Table 2:** Hazard ratios of all-cause, CVD, and cancer-related mortalities categorized by different subtypes of SLD.

Mortality	No. of cases (per 1000 person-years)	Unadjusted		Model 1		Model 2		Model 3	
		HR (95% CI)	P-value	HR (95% CI)	P-value	HR (95% CI)	P-value	HR (95% CI)	P-value
ALL-cause									
No-SLD	2277 (12.97)	ref		ref		ref		ref	
CrySLD	10 (4.44)	0.32(0.13,0.77)	**0.01**	0.36(0.16,0.80)	**0.01**	0.35(0.16,0.79)	0.01	0.46(0.20,1.05)	0.07
MASLD	852 (20.48)	1.82(1.61,2.05)	**<0.001**	1.53(1.38,1.70)	**<0.001**	1.56(1.40,1.74)	**<0.001**	1.19(1.06,1.34)	**0.006**
MetALD	60 (25.40)	2.78(1.97,3.92)	**<0.001**	2.86(1.99,4.10)	**<0.001**	2.93(2.04,4.20)	**<0.001**	2.21(1.48,3.29)	**0.004**
OtherSLD	30 (28.22)	2.98(1.76,5.07)	**<0.001**	2.92(1.69,5.03)	**0.001**	2.93(1.72,5.01)	**<0.001**	2.40(1.43,4.04)	**0.013**
CVD-related									
No-SLD	628 (4.23)	ref		ref		ref		ref	
CrySLD	1 (0.48)	0.01(0.00,0.04)	**<0.001**	0.01(0.00, 0.05)	**<0.001**	0.01(0.00, 0.05)	**<0.001**	0.01(0.00, 0.09)	**<0.001**
MASLD	240 (7.65)	2.17(1.73,2.72)	**<0.001**	1.63(1.34, 1.99)	**<0.001**	1.65(1.35, 2.02)	**<0.001**	0.96(0.78, 1.19)	0.730
MetALD	16 (9.52)	2.88(1.53,5.45)	**0.0013**	3.52(1.80, 6.89)	**0.001**	3.52(1.78, 6.96)	**0.001**	1.84(0.78, 4.33)	0.213
OtherSLD	7 (9.55)	4.02(1.70,9.53)	**0.0020**	5.81(2.33,14.47)	**0.001**	5.88(2.36,14.63)	**0.001**	4.70(2.02,10.94)	**0.002**
Cancer-related									
No-SLD	571 (3.87)	ref		ref		ref		ref	
CrySLD	4 (1.85)	0.71(0.23, 2.22)	0.56	0.73(0.25, 2.10)	0.56	0.72(0.25, 2.11)	0.55	1.01(0.34, 3.04)	0.98
MASLD	192 (6.29)	1.59(1.21, 2.09)	**0.004**	1.43(1.13, 1.81)	**0.006**	1.52(1.20, 1.93)	**0.002**	1.25(0.98, 1.59)	0.093
MetALD	14 (8.47)	2.86(1.44, 5.69)	**0.006**	3.36(1.71, 6.59)	**0.004**	3.30(1.64, 6.64)	**0.002**	2.40(1.17, 4.90)	0.08
OtherSLD	7 (9.78)	3.65(1.03,12.90)	**0.053**	3.76(1.06,13.30)	0.053	3.58(1.01,12.70)	0.067	3.81(1.02,14.27)	0.093

Abbreviations: SLD = steatotic liver disease, MASLD = metabolic dysfunctional associated fatty liver disease, OtherSLD = other specific aetiology SLD, CrySLD = cryptogenic ALD, CVD = cardiovascular disease, HR = hazard ratio. Model 1 was adjusted for age, sex and race; model 2 was adjusted for age, sex, race, drinking, smoking, hepatitis; and model 3 was further adjusted for age, sex, race, drinking, smoking, hepatitis, T2DM, Hypertension, High triglycerides, Low HDL, High C- reactive protein, obesity, tbil, alb, ast and alt. All P were FDR-adjusted.

The unadjusted cardiovascular-related mortality of the MASLD group was 2.17 times higher than that of the No-SLD group, and that of the MetALD group was 2.88 times higher than that of the No-SLD group. However, there were no differences after adjustment for demographic parameters and metabolic risk factors. The unadjusted cardiovascular-related mortality of the OtherSLD group was 4.02 times higher than that of the No-SLD group (unadjusted HR 4.02; 95% CI 1.70–9.53; *P* = 0.002), and after adjustment for potential confounders, it was 4.70 times higher (adjusted HR 4.70; 95% CI 2.02–10.94; *P* = 0.02). However, with respect to adjusted cancer-related mortality, there were no differences between the CrySLD, MASLD, MetALD, and OtherSLD groups, and the No-SLD group.

### Comparison of the baseline characteristics of participants with the various combinations of MASLD and NAFLD

Among the participants with SLD, 2,978 could be categorized as having NAFLD, and there was a moderate level of consistency between MASLD and NAFLD (Cohen’s kappa coefficient of 0.62545). Among participants with SLD, the weighted prevalence of MASLD + /NAFLD+ was 83.37%, that of MASLD + /NAFLD− was 3.16%, that of MASLD − /NAFLD+ was 7.51%, and that of MASLD − /NAFLD− was 5.96% (Fig 2, Table 3). Participants in the MASLD + /NAFLD + , MASLD + /NAFLD − , and MASLD − /NAFLD− groups were more likely to be men, had higher prevalences of diabetes, had a shorter survival time, and had higher mortality than those without SLD.

**Table 3:** Comparison of baseline characteristics of MASLD and NAFLD combinations.

Variable	Total	No-SLD	MASLD + /NAFLD+	MASLD + /NAFLD-	MASLD-/NAFLD+	MASLD-/NAFLD-	Pvalue	MASLD+ /NAFLD+ VS No-SLD	MASLD+ /NAFLD- VS No-SLD	MASLD-/NAFLD+ VS No-SLD	MASLD-/NAFLD- VS No-SLD
**Age, years**	43.42(0.39)	42.40(0.40)	47.96(0.57)	45.45(2.67)	41.79(1.45)	51.03(1.78)	**< 0.0001**	**< 0.0001**	0.255	0.682	**< 0.0001**
**Race, %**							0.53				
White	6273(85.44)	4715(84.87)	1323(87.84)	54(80.56)	93(87.34)	88(91.54)					
Black	2666(10.60)	2217(11.08)	373(8.61)	16(10.69)	39(9.68)	21(6.70)					
Other	297(3.95)	221(4.05)	64(3.55)	3(8.75)	7(2.98)	2(1.76)					
**Sex, %**							**< 0.0001**	**0.0097**	**0.0047**	0.7752	**0.00257**
Female	4401(48.55)	3313(47.10)	874(53.02)	56(74.66)	74(45.22)	84(75.75)					
Male	4835(51.45)	3840(52.90)	886(46.98)	17(25.34)	65(54.78)	27(24.25)					
**T2DM, %**							**< 0.0001**	**< 0.0001**	**0.042**	0.0501	**< 0.0001**
Normal	8235(92.92)	6609(95.26)	1349(82.46)	60(86.25)	130(97.92)	87(76.61)					
T2DM	1001(7.08)	544(4.74)	411(17.54)	13(13.75)	9(2.08)	24(23.39)					
**Hypertension, %**							**< 0.0001**	**< 0.0001**	**0.052**	**0.3**	**< 0.0001**
Normal	7620(85.00)	6097(87.83)	1283(73.41)	49(72.69)	118(84.50)	73(60.71)					
Hypertension	1616(15.00)	1056(12.17)	477(26.59)	24(27.31)	21(15.50)	38(39.29)					
**Obesity, %**							**< 0.0001**	**< 0.0001**	0.0015	0.1206	**< 0.0001**
Normal	5559(54.15)	3859(47.59)	1521(86.36)	56(70.91)	48(33.62)	75(68.64)					
Obesity	3677(45.85)	3294(52.41)	239(13.64)	17(29.09)	91(66.38)	36(31.36)					
**Smoking, %**							**< 0.0001**	**< 0.0001**	**0.0225**	0.6433	0.2227
Normal	6659(69.66)	5064(68.16)	1407(78.52)	38(46.49)	89(70.96)	61(58.91)					
Smoking	2577(30.34)	2089(31.84)	353(21.48)	35(53.51)	50(29.04)	50(41.09)					
**Hepatitis, %**							**< 0.0001**	**< 0.0001**	**< 0.0001**	**< 0.0001**	0.113
Normal	8965(97.46)	6929(97.43)	1760(100.00)	33(34.06)	139(100.00)	104(94.59)					
Hepatitis	271(2.54)	224(2.57)	0(0.00)	40(65.94)	0(0.00)	7(5.41)					
**FIB-4 score**	0.94(0.02)	0.91(0.02)	0.97(0.02)	1.85(0.74)	0.98(0.06)	1.52(0.18)	**< 0.0001**	**0.0263**	0.2118	0.3016	**0.0013**
**FIB-4 category, %**							**< 0.0001**	0.5073	**0.0017**	0.6109	**< 0.0001**
FIB-4 ≥ 2.67	178(1.39)	126(1.14)	27(1.37)	8(11.52)	2(0.78)	15(14.15)					
FIB-4 < 2.67	9058(98.61)	7027(98.86)	1733(98.63)	65(88.48)	137(99.22)	96(85.85)					
**Time, month**	300.50(2.36)	305.57(2.60)	282.66(3.27)	238.74(23.36)	310.53(6.49)	223.49(12.66)	**< 0.0001**	**< 0.0001**	**0.007**	0.4705	**< 0.0001**
**Status, %**							**< 0.0001**	**< 0.0001**	**0.0094**	0.1135	**< 0.0001**
Alive	6007(70.59)	4876(73.65)	951(58.02)	30(46.17)	108(82.73)	42(34.76)					
Death	3229(29.41)	2277(26.35)	809(41.98)	43(53.83)	31(17.27)	69(65.24)					

Abbreviations: MASLD = metabolic dysfunctional associated fatty liver disease, NAFLD = nonalcoholic fatty liver disease, MASLD + /NAFLD+ = individuals who met the definitions of MASLD and NAFLD, MASLD + /NAFLD- = those with MASLD but not NAFLD, MASLD-/NAFLD+ = those with NAFLD but not MASLD, MASLD-/NAFLD- = those with not MASLD or NAFLD, T2DM = Type 2 diabetes mellitus, FIB-4 = Fibrosis-4.

We compared the body measurement data and blood biochemical indicators of the MASLD + /NAFLD+ group with those of the other four groups (S3 Table). We found that participants in the MASLD + /NAFLD+ group were more likely to be female than those in the No-SLD group, the prevalence of type 2 diabetes was higher, they were more likely to be non-smokers, and they had higher AST and ALT activities.

### Comparison of the mortality rates associated with the various combinations of MASLD and NAFLD

There were 809, 43, 31, and 69 deaths from all causes; 226, 14, 10, and 14 cardiovascular-related deaths; and 183, 9, 6, and 19 cancer-related deaths in the MASLD + /NAFLD + , MASLD + /NAFLD − , MASLD − /NAFLD + , and MASLD − /NAFLD− groups, respectively, throughout the maximum 31-year follow-up period (median 25 years) (Table 4). When we used the No-SLD group as a reference, the MASLD + /NAFLD + , MASLD + /NAFLD − , and MASLD − /NAFLD− groups were at higher risks of all-cause mortality (unadjusted HR, 1.79; 95% CI 1.58–2.02; *P* < 0.0001; unadjusted HR, 2.83; 95% CI 1.52–5.28; *P* = 0.001; and unadjusted HR, 3.86; 95% CI 2.65–5.61; *P* < 0.0001; respectively; Table 4). After adjustment for potential confounders, the MASLD + /NAFLD+ and MASLD − /NAFLD− groups retained higher risks of all-cause mortality. However, no differences in the risk of cardiovascular mortality were identified among the groups after adjustment for potential confounders. Adjustment for physical activity levels and poverty-income ratio, these results are consistent with the aforementioned findings (S4 Table). Finally, the cancer-related mortality rate of the MASLD − /NAFLD− group was 4.32 times higher than that of the No-SLD group (adjusted HR, 4.32; 95% CI 1.97–9.46; *P* = 0.004), but that of the other three groups did not differ.

**Table 4:** Comparison of combined mortality rates between MASLD and NAFLD.

Mortality	No. of cases (per 1000 person-years)	Unadjusted		Model 1		Model 2		Model 3	
		HR (95% CI)	P-value	HR (95% CI)	P-value	HR (95% CI)	P-value	HR (95% CI)	P-value
ALL-cause									
No-SLD	2277 (12.97)	ref		ref		ref		ref	
MASLD + /NAFLD+	809 (20.19)	1.79(1.58,2.02)	**<0.001**	1.52(1.35,1.70)	**<0.001**	1.57(1.40,1.77)	**<0.001**	1.21(1.07,1.37)	**0.006**
MASLD + /NAFLD-	43 (28.15)	2.83(1.52,5.28)	**0.0013**	1.92(0.97,3.77)	0.08	1.36(0.69,2.68)	0.38	0.90(0.51,1.59)	0.71
MASLD-/NAFLD+	31 (8.96)	0.65(0.36,1.17)	0.15	0.74(0.41,1.30)	0.29	0.75(0.42,1.32)	0.38	0.82(0.48,1.40)	0.61
MASLD-/NAFLD-	69 (31.11)	3.86(2.65,5.61)	**<0.001**	3.57(2.53,5.03)	**<0.001**	3.57(2.53,5.05)	**<0.001**	2.69(1.77,4.07)	**<0.001**
CVD-related									
No-SLD	628 (4.23)	ref		ref		ref		ref	
MASLD + /NAFLD+	226 (7.46)	2.11(1.67, 2.66)	**<0.001**	1.61(1.31, 1.98)	**<0.001**	1.63(1.32, 2.01)	**<0.001**	0.94(0.74, 1.19)	0.73
MASLD + /NAFLD-	14 (13.32)	4.25(1.49,12.14)	**0.013**	2.16(0.51, 9.16)	0.387	2.25(0.44,11.53)	0.44	1.25(0.35, 4.54)	0.73
MASLD-/NAFLD+	10 (3.18)	0.81(0.31, 2.08)	0.66	1.05(0.41, 2.71)	0.92	1.05(0.40, 2.76)	0.92	1.23(0.48, 3.19)	0.73
MASLD-/NAFLD-	14 (10.33)	3.96(1.90, 8.27)	**0.002**	4.95(2.46, 9.97)	**<0.001**	4.77(2.36, 9.62)	**<0.001**	2.22(0.85, 5.81)	0.40
Cancer-related									
No-SLD	571 (3.87)	ref		ref		ref		ref	
MASLD + /NAFLD+	183 (6.18)	1.58(1.19,2.09)	**0.002**	1.44(1.13, 1.84)	**0.008**	1.53(1.20, 1.95)	**0.002**	1.27(1.00, 1.63)	0.1
MASLD + /NAFLD-	9 (9.53)	1.87(0.64,5.42)	0.29	1.23(0.34, 4.49)	0.75	1.41(0.36, 5.48)	0.62	1.06(0.30, 3.79)	0.92
MASLD-/NAFLD+	6 (1.94)	0.61(0.24,1.53)	0.29	0.65(0.27, 1.56)	0.44	0.66(0.27, 1.57)	0.46	0.76(0.33, 1.75)	0.69
MASLD-/NAFLD-	19 (13.27)	5.18(2.75,9.79)	**<0.001**	5.72(3.00,10.88)	**<0.001**	5.24(2.67,10.29)	**<0.001**	4.32(1.97, 9.46)	**0.004**

Abbreviations: MASLD = metabolic dysfunctional associated fatty liver disease, NAFLD = nonalcoholic fatty liver disease, CVD = cardiovascular disease, HR = hazard ratio.

Model 1 was adjusted for age, sex and race; model 2 was adjusted for age, sex, race, smoking; and model 3 was further adjusted for age, sex, race, smoking, T2DM, Hypertension, obesity, tbil, alb, ast, alt.

All P were FDR-adjusted.

### Risk factors for all-cause, cardiovascular-related, and cancer-related mortality in participants with MASLD and NAFLD

We identified similar risk factors for all-cause mortality in participants with MASLD or NAFLD (Table 5). After adjustment for ethnicity, sex, age, and smoking and alcohol consumption status, we found that diabetes, hypertension, obesity, central obesity, and high fibrosis score were the risk factors for all-cause mortality in participants with MASLD or NAFLD. Furthermore, high CRP concentration was found to be risk factors for all-cause mortality in participants with NAFLD. High fibrosis score was the most substantial risk factor for all-cause mortality in participants with MASLD, whereas high CRP concentration was the most substantial risk factor for all-cause mortality in participants with NAFLD, followed by type 2 diabetes. In addition, diabetes and hypertension were found to be risk factors for cardiovascular-related mortality in participants with either MASLD or NAFLD; central obesity was a risk factor in those with MASLD; high TG concentration and obesity were risk factors in those with NAFLD. Finally, hepatitis was found to be a predictor of cancer-related mortality in MASLD and low HDL-C concentration was a predictor of cancer-related mortality in NAFLD.

**Table 5:** Hazard ratios of risk factors for all- cause and cause- specific mortality among the MASLD+ and among the NAFLD + : NHANES III.

Risk factors	All-cause mortality				CVD-specific mortality				Cancer-specific mortality			
	MASLD		NAFLD		MASLD		NAFLD		MASLD		NAFLD	
	HR (95%CI)	P	HR (95%CI)	P	HR (95%CI)	P	HR (95%CI)	P	HR (95%CI)	P	HR (95%CI)	P
**T2DM**	1.61(1.33,1.95)	**<0.0001**	1.67(1.41,1.99)	**<0.0001**	2.11(1.52, 2.92)	**<0.0001**	2.26(1.69,3.03)	**<0.0001**	1.00(0.58,1.72)	1	1.10(0.69,1.76)	0.69
**High Homair**	1.21(0.94,1.56)	0.15	1.30(1.08,1.56)	**0.005**	1.36(0.87, 2.15)	0.18	1.62(1.21,2.17)	**0.001**	1.41(0.86,2.32)	0.17	1.24(0.85,1.81)	0.27
**Hypertension**	1.47(1.11,1.95)	**0.01**	1.66(1.33,2.06)	**<0.0001**	2.00(1.20, 3.33)	**0.01**	2.48(1.75,3.52)	**<0.0001**	1.07(0.68,1.69)	0.76	1.43(0.98,2.10)	0.07
**High triglycerides**	1.07(0.87,1.31)	0.54	1.15(0.94,1.42)	0.18	1.23(0.84, 1.81)	0.28	1.40(1.00,1.98)	**0.05**	1.10(0.62,1.97)	0.75	1.23(0.71,2.11)	0.46
**High C- reactive protein**	1.61(0.94,2.73)	0.08	1.84(1.19,2.83)	**0.01**	1.11(0.62, 1.99)	0.72	1.56(0.93,2.61)	0.09	0.27(0.04,1.86)	0.18	0.79(0.22,2.83)	0.72
**Obesity**	0.68(0.47,0.96)	**0.03**	0.62(0.46,0.83)	**0.001**	0.75(0.35, 1.61)	0.46	0.46(0.27,0.78)	**0.004**	0.57(0.29,1.13)	0.11	0.54(0.28,1.05)	0.07
**Low HDL**	1.06(0.87,1.29)	0.59	1.14(0.93,1.38)	0.2	0.91(0.62, 1.33)	0.63	0.95(0.71,1.27)	0.73	0.68(0.13,3.73)	0.66	1.63(1.04,2.56)	**0.03**
**Central obesity**	1.67(1.18,2.37)	**0.004**	1.47(1.04,2.07)	**0.03**	2.16(1.07, 4.38)	**0.03**	1.85(0.91,3.76)	0.09	1.31(0.74,2.34)	0.36	1.23(0.61,2.46)	0.56
**High- risk fibrosis**	1.73(1.11,2.71)	**0.02**	1.51(1.02,2.23)	**0.04**	1.40(0.68, 2.89)	0.37	1.47(0.68,3.16)	0.33	1.24(0.61,2.54)	0.55	1.87(0.71,4.91)	0.21
**Hepatitis**	1.84(0.73,4.62)	0.19			2.37(0.29,19.37)	0.42			2.59(1.37,4.89)	**0.003**		

Abbreviations: MASLD = metabolic dysfunctional associated fatty liver disease, NAFLD = nonalcoholic fatty liver disease, HTN = Hypertension, TG = triglyceride, CRP = C-reactive protein, HDL = High-density lipoprotein. HRs and 95% CI were weighted to US population and adjusted for race, gender, age, smoking and drinking. High- risk fibrosis: Defined as FIB-4 ≥ 2.67.

## Discussion

In the present study, we compared the clinical characteristics and all-cause, CVD-related, and cancer-related mortality rates of patients with four distinct subtypes of SLD and four groups of patients who met the various diagnostic criteria for MASLD and/or NAFLD; and identified the risk factors for all-cause, CVD-related, and cancer-related mortality in patients with NAFLD or MASLD. Firstly, we noted that SLD is quite common in the US and that MASLD is the most prevalent type (weighted percentage 86.64%). We also found that OtherSLD is associated with the highest risk of all-cause mortality, followed by MetALD and finally MASLD. Secondly, as expected, there was a moderate level of consistency between the definitions of MASLD and NAFLD (kappa = 0.625), with 83.37% of patients with SLD meeting the criteria for both syndromes. We found no difference in the higher risks of all-cause mortality *vs*. the No-SLD group between the MASLD + /NAFLD+ and MASLD + /NAFLD− groups. Thirdly, the risk factors for all-cause mortality and cardiovascular mortality differed between the MASLD and NAFLD groups. High fibrosis score was found to be a predictor of all-cause mortality in MASLD and high CRP concentration were the predictors of all-cause mortality in NAFLD.

The present study is one of the few studies to have reported the prevalences of the various subtypes of SLD to date. The recent guidelines recommended changing the name of the condition from NAFLD to MASLD [9], and MASLD remains the predominant chronic liver disease after this name change. In our study, MASLD accounted for 86.54% of SLD patients. Consistent with several other studies, MASLD was the most prevalent subtype among SLD patients [17,18]. In making this suggestion, the authors took full account of the effect of alcohol on SLD, and divided patients into those with MASLD, MetALD, or ALD, subsequently, ALD was categorized as OtherSLD. The present findings have confirmed the clinical importance of this distinction. Over a prolonged follow-up period of a median of 25 years, MASLD, MetALD, OtherSLD and MASLD-/NAFLD- were associated with higher risks of all-cause mortality, and OtherSLD was found to be associated with the highest risk of death, followed by MetALD and MASLD. Given that the principal group of patients included in OtherSLD, MASLD-/NAFLD- are those with ALD, alcohol abuse is likely to be the most harmful etiologic factor in patients with SLD. By 2018, approximately 2.3 billion people were drinking alcohol regularly worldwide, and the vast majority (more than 90%) of these people had fatty liver disease [19]. Frequent alcohol consumption increases the risks of steatohepatitis and liver fibrosis, which can lead to cirrhosis and liver cancer, and increases the risk of alcohol-induced liver disease-related mortality 168-fold [20]. Indeed, approximately one-fourth of people with fatty liver die because of cirrhosis and one-fifth die because of hepatocellular carcinoma [21]. In addition, although the adjusted HR for cardiovascular mortality in OtherSLD was 4.70 (95% CI 2.02–10.94), only 7 cardiovascular deaths occurred in this subgroup. This small event counting may lead to inaccurate effect estimates (wide confidence intervals) and potential risk overestimation. These findings need to be validated in larger queues with sufficient event rates to distinguish between true associations and random variability.

It is worth noting that the new set of clinical disease guidelines have been created a new definition of MetALD, defining a group of patients that was once often overlooked [9]. Although there is a consensus among researchers regarding the harms of alcohol misuse, determining the long-term risk of the fatty liver induced by moderate alcohol consumption (MAC) had been difficult, because there was no standard definition of the alcohol consumption required to induce MAC [21]. Previous studies have shown that MAC reduces the risk of SLD, and Sookoian *et al* [22,23]. showed that MAC (associated with the consumption of <40 g/d alcohol) is significantly more protective against NAFLD in women than in men and that this effect is not affected by body mass. However, a Mendelian randomization analysis showed that MAC does not have a positive effect on NAFLD [24]. Chang *et al* [25]. found that moderate alcohol consumption (10–29.9 g/d in men and 10–19.9 g/d in women) promotes hepatic scarring, fibrosis, and end-stage cirrhosis in patients with NAFLD. Notably, MetALD patients exhibited significantly higher SBP and DBP than MASLD counterparts, suggesting a synergistic interaction between alcohol-related hepatotoxicity and metabolic dysfunction in exacerbating cardiovascular strain [26]. These elevations may reflect heightened systemic inflammation or endothelial dysfunction in MetALD. Furthermore, liver damage is significantly more severe in light and moderate drinkers in the presence of metabolic risk factors [27]. In the present study, we found that the all-cause mortality rate of patients with MetALD was 2.21 times higher than that of patients without SLD, suggesting that MAC may increase all-cause mortality in patients with fatty liver, and in another study, patients with MetALD and metabolic dysfunction had a worse prognosis [17].

After adjusting for age, sex, ethnicity, smoking, alcohol consumption, hepatitis, and metabolic risk factors (including diabetes, hypertension, hypertriglyceridemia, elevated CRP, obesity, and increased bilirubin, ALP, AST, and ALT), MASLD remained significantly associated with all-cause mortality (adjusted HR = 1.19; P = 0.006). However, this adjusted risk was substantially attenuated compared to the unadjusted association (HR = 1.82). MASLD was described as a metabolic disease that is characterized by hepatic steatosis and associated with cardiovascular risk; and steatosis alone can readily progress to non-alcoholic steatohepatitis (NASH) and advanced fibrosis [28]. Likewise, MASLD is associated with cardiometabolic risk, and metabolic dysregulation, indicated by the presence of obesity and T2DM, might hasten the progression of NASH to cirrhosis [29]. Indeed, the larger the number of components of the metabolic syndrome a patient with fatty liver disease has, the higher their risk of death [30]. The study by Li *et al* [17]. Using NHANESIII data showed that MASLD *per se* is not associated with higher all-cause mortality, which is most likely due to the fact that they used a distinct list of confounders, which included BMI. However, previous studies have shown a U-shaped relationship between BMI and mortality in patients with NAFLD [31], and many have shown that the absence of obesity is associated with a higher risk of death than obesity [32,33]. Therefore, we further categorized continuous variables and adjusted for the obesity indicator instead of BMI in the analysis. The present model was capable of determining whether metabolic dysregulation or MASLD *per se* principally contributes to the higher risk of mortality in patients with MASLD.

The moderate agreement between MASLD and NAFLD participants arises from fundamental differences in their diagnostic frameworks: MASLD emphasizes positive identification of metabolic dysfunction, whereas NAFLD primarily relies on excluding viral hepatitis and excessive alcohol consumption. Because specific treatments are available for neither MASLD nor NAFLD, we focused on analyzing the risk factors for all-cause mortality for patients with MASLD or NAFLD. The predictors for all-cause mortality were similar for those with MASLD or NAFLD, and included diabetes, hypertension, obesity, central obesity, and severe liver fibrosis. Notably, a high risk of fibrosis was the most significant predictor for patients with MASLD, whereas this was a high CRP concentration for those with NAFLD, followed by diabetes. These findings may indicate that the definition of NAFLD may better reflect the inflammation that is characteristic of the disease than that of MASLD. Indeed, inflammatory cytokines are recognized to be important contributors to the development and progression of NAFLD [34], and continuous inflammation promotes the progression of alcoholic steatohepatitis and NASH [35]. In patients with MASLD, hepatitis was not found to be a risk factor for all-cause mortality, and therefore the findings of the present study are consistent with the guidelines that allow the coexistence of MASLD and hepatitis, but the previous diagnostic criteria for NAFLD also excluded hepatitis [36]. Given the current absence of a specific drug treatment, strategies aimed at ameliorating the metabolic dysregulation involved in MASLD are important. In the future, systematic screening for metabolic risks should be considered in patients with suspected chronic liver disease, which should permit a personalized treatment approach.

The present study had the following limitations. First, a diagnosis of SLD was made using ultrasonography rather than liver biopsy, because the latter is invasive, impractical, and unethical in epidemiologic studies. However, the ultrasonographic data from NHANESIII was reevaluated to confirm the diagnosis of hepatic steatosis. Second, the unavailability of fasting and 2-hour postprandial glucose measurements in NHANES III due to high rates of missing data constrained the comprehensiveness of our T2DM definition. Although we supplemented HbA1c ≥ 6.5% with physician-diagnosed diabetes and glucose-lowering medication use, future studies incorporating standardized glycemic measurements are needed for prospective validation of these findings. Third, we used the Fibrosis-4(FIB-4) test rather than liver biopsy to grade liver fibrosis, for the same reasons given above. Given the substantial overlap between individuals with MASLD and NAFLD and the absence of a specific test for MASLD, the non-invasive FIB-4 test is a well-validated and accepted method for the diagnosis of advanced fibrosis in individuals with NAFLD [16]. Fourth, alcohol consumption was determined via self-report, which is susceptible to potential underreporting due to social desirability bias. Such misclassification may have compromised the accuracy of MASLD and MetALD subtype classifications. In addition, the sample sizes for the Other-SLD and cryptogenic SLD subgroups were small, which substantially limited the statistical power of our analyses. This increases the risk of Type II errors, meaning that the non-significant associations observed for these subgroups should not be interpreted as evidence of no elevated mortality risk, but rather as findings that require validation in larger, specifically designed cohorts. Finally, while the baseline data for this study were derived from the NHANES III cohort (1988–1994), it is undeniable that both disease spectra and biochemical profiles have evolved substantially over the past three decades. Nevertheless, in the context of the ongoing obesity epidemic, contemporary patients experience earlier disease onset than the NHANES III cohort, yet exhibit similar or more adverse metabolic characteristics. Thus, our findings provide critical insights into the long-term risks facing patients in the current era of pervasive metabolic dysfunction.

In summary, we used the NHANESIII dataset to compare the clinical characteristics, all-cause mortality rate, and specific mortality rates of several subgroups of individuals with SLD. We found that OtherSLD is associated with the highest risk of all-cause mortality, followed by MetALD and MASLD. This suggests that using alcohol consumption as the primary criterion for placing SLD patients into MASLD, MetALD, and OtherSLD groups is a useful means of differentiating the risk of death among patients with SLD. In addition, we found that liver fibrosis is the most significant risk factor for all-cause mortality in patients with MASLD, and that high CRP concentration is the most significant risk factor for all-cause mortality in those with NAFLD. Therefore, despite the huge overlap between patients with MASLD and those with NAFLD, the pathogenic mechanisms are somewhat distinct. Furthermore, we found that the higher risk of all-cause mortality in patients with MASLD is not related to sociodemographic or metabolic factors, which implies that the risks associated with MASLD are predominantly linked to the liver lesions themselves.

## Supporting information

S1 Table Comparison of continuous variables in baseline characteristics of subjects with different subtypes of SLD.
(DOCX)

S2 Table Hazard ratios for all-cause, cardiovascular disease, and cancer-related mortality classified by different subtypes of SLD, further adjusted for physical activity and poverty-income ratio.
(DOCX)

S3 Table Comparison of continuous variables in baseline characteristics between MASLD and NAFLD cohorts.
(DOCX)

S4 Table Comparison of mortality between MASLD and NAFLD cohorts, further adjusted for physical activity and poverty-income ratio.
(DOCX)

## Abbreviation:

NAFLD: nonalcoholic fatty liver disease
CVD: cardiovascular disease
MASLD: metabolic dysfunction-associated steatotic liver disease
SLD: steatotic liver disease
ALD: alcohol-related liver disease
NHANES: national health and nutrition examination survey
BMI: body mass index
WHR: waist-to-hip ratio
SBP: systolic blood pressure
DBP: diastolic blood pressure
CRP: C-reactive protein
HbA1c: glycosylated hemoglobin
BG: blood glucose
INS: insulin
AST: aspartate aminotransferase
ALT: alanine transaminase
TC: total cholesterol
TG: triglyceride
ALB: albumin
TBil: total bilirubin
HDL-C: high-density lipoprotein-cholesterol
BUN: blood urea nitrogen
Scr: serum creatinine
CP: C-peptide
UA: uric acid
ALP: alkaline phosphatase
CMRF: cardiometabolic risk factor
OtherSLD: other specific aetiology SLD
CrySLD: cryptogenic ALD
T2DM: Type 2 diabetes mellitus
HRs: hazard ratios
CIs: confidence intervals
MAC: moderate alcohol consumption
NASH: non-alcoholic steatohepatitis
FIB-4: Fibrosis-4
