# Supplementary material for: Comparative mortality outcomes in metabolic dysfunction-associated steatotic liver disease and nonalcoholic fatty liver disease subtypes in the United States
Source: PLoS One. 2025 Oct 31;20(10):e0335230. doi: 10.1371/journal.pone.0335230 (PMC12578175; doi:10.1371/journal.pone.0335230)
Supplement: S1 Table — Comparison of continuous variables in baseline characteristics of subjects with different subtypes of SLD. (DOCX) [file pone.0335230.s001.docx]

**S1 Table** Comparison of continuous variables in baseline characteristics of subjects with different subtypes of SLD.

| **Variable** | **Total** | **No-SLD** | **CrySLD** | **MASLD** | **MetALD** | **OtherSLD** | **Pvalue** | **CrySLD VS**  **No-SLD** | **MASLD vs**  **No-SLD** | **MetALD vs**  **No-SLD** | **OtherSLD vs**  **No-SLD** |
| --- | --- | --- | --- | --- | --- | --- | --- | --- | --- | --- | --- |
| **TC, mmol/L** | 5.25(0.02) | 5.18(0.02) | 4.69(0.12) | 5.53(0.04) | 5.94(0.15) | 5.90(0.37) | **< 0.0001** | **0.000357** | **< 0.001** | **< 0.001** | 0.06477 |
| **TG, mmol/L** | 1.57(0.03) | 1.39(0.02) | 0.91(0.04) | 2.38(0.06) | 1.95(0.13) | 3.68(1.95) | **< 0.0001** | **< 0.001** | **< 0.001** | **< 0.001** | 0.244 |
| **HDL-C, mmol/L** | 1.31(0.01) | 1.35(0.01) | 1.50(0.06) | 1.13(0.01) | 1.35(0.06) | 1.58(0.09) | **< 0.0001** | **0.0203** | **< 0.001** | 0.9508 | **0.0113** |
| **CRP, mg/dL** | 0.39(0.01) | 0.37(0.01) | 0.28(0.05) | 0.48(0.02) | 0.40(0.04) | 0.29(0.03) | **< 0.0001** | **0.0486** | **< 0.001** | 0.4374 | **0.0132** |
| **UA, μmol/L** | 313.48(1.26) | 303.61(1.07) | 249.81(15.80) | 357.48(3.31) | 372.21(9.37) | 374.58(13.25) | **< 0.0001** | **0.00143** | **< 0.001** | **< 0.001** | **< 0.001** |
| **BUN, mmol/L** | 13.90(0.11) | 13.74(0.12) | 12.85(0.63) | 14.66(0.22) | 14.76(0.66) | 13.38(0.86) | **0.001** | 0.172 | **< 0.001** | 0.133 | 0.675 |
| **TBiL, mg/dL** | 0.62(0.01) | 0.62(0.01) | 0.62(0.06) | 0.61(0.01) | 0.67(0.06) | 0.80(0.12) | 0.57 |  |  |  |  |
| **Scr, μmol/L** | 93.70(0.28) | 93.20(0.34) | 88.46(2.93) | 95.98(0.61) | 96.96(2.27) | 98.43(3.88) | **< 0.001** | 0.115664 | **< 0.001** | 0.095652 | 0.185811 |
| **AST, U/L** | 21.35(0.21) | 20.25(0.19) | 21.54(1.46) | 25.58(0.69) | 28.18(1.93) | 37.41(3.75) | **< 0.0001** | 0.38704 | **< 0.001** | **< 0.001** | **< 0.001** |
| **ALT, U/L** | 17.73(0.45) | 15.91(0.35) | 16.58(1.80) | 25.49(1.02) | 24.50(2.03) | 30.80(3.37) | **< 0.0001** | 0.707656 | **< 0.001** | **< 0.001** | **< 0.001** |
| **ALB, g/L** | 42.18(0.20) | 42.23(0.21) | 42.14(0.38) | 41.94(0.23) | 42.50(0.44) | 42.50(0.73) | 0.18 |  |  |  |  |
| **HbA1c, %** | 5.30(0.02) | 5.22(0.02) | 5.05(0.05) | 5.68(0.05) | 5.56(0.15) | 5.36(0.14) | **< 0.0001** | **0.00163** | **< 0.001** | **0.03083** | 0.35686 |
| **BG, mmol/L** | 5.41(0.02) | 5.28(0.03) | 4.98(0.09) | 5.98(0.07) | 6.18(0.34) | 6.20(0.45) | **< 0.0001** | **< 0.001** | **< 0.001** | **0.011793** | 0.05077 |
| **C-P, nmol/L** | 0.68(0.01) | 0.58(0.01) | 0.46(0.05) | 1.09(0.02) | 0.97(0.07) | 0.84(0.09) | **< 0.0001** | **0.02751** | **< 0.001** | **< 0.001** | **0.00417** |
| **INS, pmol/L** | 10.77(0.28) | 9.22(0.18) | 7.25(0.65) | 18.02(0.81) | 13.48(1.52) | 11.87(1.63) | **< 0.0001** | **0.00413** | **< 0.001** | **0.00721** | 0.10792 |
| **SBP, mmHg** | 120.72(0.35) | 119.01(0.33) | 107.68(1.40) | 128.18(0.56) | 134.59(2.57) | 132.90(3.11) | **< 0.0001** | **< 0.001** | **< 0.001** | **< 0.001** | **< 0.001** |
| **DBP, mmHg** | 74.17(0.19) | 73.24(0.23) | 69.59(1.53) | 78.09(0.41) | 81.07(1.23) | 81.41(2.61) | **< 0.0001** | **0.02653** | **< 0.001** | **< 0.001** | **0.00355** |
| **BMI,** **kg/m^2^** | 26.49(0.12) | 25.54(0.10) | 21.34(0.61) | 30.95(0.31) | 30.06(0.82) | 27.81(1.53) | **< 0.0001** | **< 0.001** | **< 0.001** | **< 0.001** | 0.149 |
| **Waist, cm** | 91.62(0.31) | 88.92(0.27) | 75.70(1.80) | 104.13(0.70) | 103.53(1.88) | 98.54(3.47) | **< 0.0001** | **< 0.001** | **< 0.001** | **< 0.001** | **0.00791** |
| **WHR** | 0.91(0.00) | 0.89(0.00) | 0.83(0.02) | 0.97(0.00) | 0.99(0.02) | 0.97(0.01) | **< 0.0001** | **0.000897** | **< 0.001** | **< 0.001** | **< 0.001** |

Abbreviations: SLD = steatotic liver disease, MASLD = metabolic dysfunctional associated fatty liver disease, OtherSLD = other specific aetiology SLD, CrySLD = cryptogenic ALD, TC = total cholesterol, TG = triglyceride, HDL-C = High-density lipoprotein cholesterol, CRP = C-reactive protein, UA = uric acid, BUN = blood urea nitrogen, TBiL = total bilirubin, Scr = blood creatinine, AST = aspartate amino-transferase, ALT = alanine aminotransferase, ALB = albumin, HbA1c = glycosylated hemoglobin, BG = blood glucose, C-P = C-Peptide, INS = insulin, SBP = systolic blood pressure, DBP = diastolic blood pressure, BMI = body mass index, WHR = waist-to-hip ratio, T2DM = Type 2 diabetes mellitus, FIB-4 = Fibrosis-4.
