# Supplementary material for: Comparative mortality outcomes in metabolic dysfunction-associated steatotic liver disease and nonalcoholic fatty liver disease subtypes in the United States
Source: PLoS One. 2025 Oct 31;20(10):e0335230. doi: 10.1371/journal.pone.0335230 (PMC12578175; doi:10.1371/journal.pone.0335230)
Supplement: S2 Table — Hazard ratios for all-cause, cardiovascular disease, and cancer-related mortality classified by different subtypes of SLD, further adjusted for physical activity and poverty-income ratio. (DOCX) [file pone.0335230.s002.docx]

**S2 Table** Hazard ratios for all-cause, cardiovascular disease, and cancer-related mortality classified by different subtypes of SLD, further adjusted for physical activity and poverty-income ratio.

| **Mortality** | **No. of Cases** | **Unadjusted** | | **Adjusted✝** | |
| --- | --- | --- | --- | --- | --- |
|  |  | **HR (95% CI)** | **P-value** | **HR (95% CI)** | **P-value** |
| ALL-cause |  |  |  |  |  |
| No-SLD | 2277 | ref |  | ref |  |
| CrySLD | 10 | 0.32(0.13,0.77) | **0.01** | 0.47(0.21,1.08) | **0.07** |
| MASLD | 852 | 1.82(1.61,2.05) | **<0.001** | 1.17(1.04,1.32) | **0.013** |
| MetALD | 60 | 2.78(1.97,3.92) | **<0.001** | 2.17(1.46,3.22) | **0.004** |
| OtherSLD | 30 | 2.98(1.76,5.07) | **<0.001** | 2.28(1.32,3.93) | **0.006** |
| CVD-related |  |  |  |  |  |
| No-SLD | 628 | ref |  | ref |  |
| CrySLD | 1 | 0.01(0.00,0.04) | **<0.001** | 0.01(0.00, 0.09) | **0.0004** |
| MASLD | 240 | 2.17(1.73,2.72) | **<0.001** | 0.94(0.76, 1.17) | 0.59 |
| MetALD | 16 | 2.88(1.53,5.45) | **0.0013** | 1.91(0.85, 4.33) | 0.16 |
| OtherSLD | 7 | 4.02(1.70,9.53) | **0.0020** | 4.42(1.84,10.65) | **0.002** |
| Cancer-related |  |  |  |  |  |
| No-SLD | 571 | ref |  | ref |  |
| CrySLD | 4 | 0.71(0.23, 2.22) | 0.56 | 1.01(0.33, 3.08) | 0.98 |
| MASLD | 192 | 1.59(1.21, 2.09) | **0.004** | 1.23(0.97, 1.57) | 0.12 |
| MetALD | 14 | 2.86(1.44, 5.69) | **0.006** | 2.37(1.15, 4.87) | 0.08 |
| OtherSLD | 7 | 3.65(1.03,12.90) | **0.053** | 3.59(0.92,14.05) | 0.12 |

Abbreviations: SLD = steatotic liver disease, MASLD = metabolic dysfunctional associated fatty liver disease, OtherSLD = other specific aetiology SLD, CrySLD = cryptogenic ALD, CVD = cardiovascular disease, HR= hazard ratio. All P were FDR-adjusted.

**✝**Model was adjusted for age, sex, race, drinking, smoking, hepatitis, T2DM, Hypertension, High triglycerides, Low HDL, High C- reactive protein, obesity, tbil, alb, ast, alt, physical activity and poverty-income ratio (PIR).

Physical activity: The inactive group was defined as individuals reporting no leisure-time physical activity.The active group comprised those meeting recommended physical

activity levels : either ≥5 sessions per week of self-reported moderate leisure-time activity (MET 3–6) or ≥3 sessions per week of vigorous leisure-time activity (MET ≥6).

The insufficiently active group included participants not classified as inactive but failing to meet the recommended physical activity criteria.

PIR was categorized as low (≤1.30; reference) or high (>1.3).
