# Supplementary material for: Comparative mortality outcomes in metabolic dysfunction-associated steatotic liver disease and nonalcoholic fatty liver disease subtypes in the United States
Source: PLoS One. 2025 Oct 31;20(10):e0335230. doi: 10.1371/journal.pone.0335230 (PMC12578175; doi:10.1371/journal.pone.0335230)
Supplement: S3 Table — Comparison of continuous variables in baseline characteristics between MASLD and NAFLD cohorts. (DOCX) [file pone.0335230.s003.docx]

**S3 Table** Comparison of continuous variables in baseline characteristics between MASLD and NAFLD cohorts.

| **Variable** | **Total** | **No-SLD** | **MASLD+**  **/NAFLD+** | **MASLD+**  **/NAFLD-** | **MASLD-**  **/NAFLD+** | **MASLD-**  **/NAFLD-** | **Pvalue** | **MASLD+/NAFLD+**  **vs**  **No-SLD** | **MASLD+/NAFLD-**  **vs**  **No-SLD** | **MASLD-/NAFLD+**  **vs**  **No-SLD** | **MASLD-/NAFLD-**  **Vs**  **No-SLD** |
| --- | --- | --- | --- | --- | --- | --- | --- | --- | --- | --- | --- |
| **TC, mmol/L** | 5.25(0.02) | 5.18(0.02) | 5.55(0.05) | 4.98(0.28) | 5.23(0.17) | 5.82(0.21) | **< 0.0001** | **< 0.0001** | 0.4618 | 0.7744 | **0.00364** |
| **TG, mmol/L** | 1.57(0.03) | 1.39(0.02) | 2.38(0.06) | 2.30(0.29) | 1.32(0.12) | 2.61(0.80) | **< 0.0001** | **< 0.0001** | **0.003** | 0.6102 | 0.1311 |
| **HDL-C,mmol/L** | 1.31(0.01) | 1.35(0.01) | 1.13(0.01) | 1.05(0.05) | 1.46(0.05) | 1.42(0.05) | **< 0.0001** | **< 0.0001** | **< 0.0001** | **0.036** | 0.155 |
| **CRP, mg/dL** | 0.39(0.01) | 0.37(0.01) | 0.48(0.02) | 0.38(0.07) | 0.31(0.03) | 0.38(0.04) | **< 0.001** | **< 0.0001** | 0.8733 | 0.0789 | 0.8245 |
| **UA, μmol/L** | 313.48(1.26) | 303.61(1.07) | 357.36(3.28) | 360.64(15.79) | 301.19(14.64) | 365.30(10.65) | **< 0.0001** | **< 0.0001** | **0.0007** | 0.8693 | **< 0.0001** |
| **BUN, mmol/L** | 13.90(0.11) | 13.74(0.12) | 14.67(0.22) | 14.39(0.73) | 13.55(0.48) | 14.21(0.70) | **< 0.001** | **< 0.0001** | 0.366 | 0.675 | 0.501 |
| **TBiL, mg/dL** | 0.62(0.01) | 0.62(0.01) | 0.60(0.01) | 0.72(0.13) | 0.60(0.05) | 0.77(0.08) | **0.03** | 0.1485 | 0.4323 | 0.6487 | 0.0828 |
| **Scr, μmol/L** | 93.70(0.28) | 93.20(0.34) | 95.75(0.52) | 102.13(7.34) | 92.68(2.34) | 96.18(2.84) | **0.01** | **0.0005** | 0.2293 | 0.8230 | 0.302173 |
| **AST, U/L** | 21.35(0.21) | 20.25(0.19) | 24.95(0.54) | 42.12(8.57) | 23.85(1.28) | 32.06(2.33) | **< 0.0001** | **< 0.0001** | **0.0142** | **0.0097** | **< 0.0001** |
| **ALT, U/L** | 17.73(0.45) | 15.91(0.35) | 25.00(0.98) | 38.36(5.25) | 19.03(1.30) | 27.63(2.29) | **< 0.0001** | **< 0.0001** | **0.0001** | **0.0277** | **< 0.0001** |
| **ALB, g/L** | 42.18(0.20) | 42.23(0.21) | 41.99(0.21) | 40.43(1.40) | 42.36(0.30) | 42.39(0.57) | 0.09 |  |  |  |  |
| **HbA1c, %** | 5.30(0.02) | 5.22(0.02) | 5.68(0.05) | 5.64(0.31) | 5.15(0.05) | 5.58(0.18) | **< 0.0001** | **< 0.0001** | 0.1809 | 0.2257 | **0.0485** |
| **BG, mmol/L** | 5.41(0.02) | 5.28(0.03) | 5.98(0.07) | 6.12(0.44) | 5.16(0.07) | 6.53(0.40) | **< 0.0001** | **< 0.0001** | 0.0687 | 0.0690 | **0.00339** |
| **C-P, nmol/L** | 0.68(0.01) | 0.58(0.01) | 1.09(0.02) | 1.32(0.12) | 0.62(0.05) | 0.96(0.07) | **< 0.0001** | **< 0.0001** | **< 0.0001** | 0.542 | **< 0.0001** |
| **INS, pmol/L** | 10.77(0.28) | 9.22(0.18) | 17.83(0.84) | 23.01(3.06) | 9.21(0.72) | 13.25(1.62) | **< 0.0001** | **< 0.0001** | **< 0.0001** | 0.991 | **0.015** |
| **SBP, mmHg** | 120.72(0.35) | 119.01(0.33) | 128.23(0.56) | 126.64(2.91) | 116.66(2.08) | 135.11(2.36) | **< 0.0001** | **< 0.0001** | **0.0109** | 0.2779 | **< 0.0001** |
| **DBP, mmHg** | 74.17(0.19) | 73.24(0.23) | 78.13(0.37) | 77.02(3.38) | 74.20(1.19) | 80.73(1.60) | **< 0.0001** | **< 0.0001** | 0.277 | 0.46 | **< 0.0001** |
| **BMI, kg/m^2^** | 26.49(0.12) | 25.54(0.10) | 31.00(0.31) | 29.52(0.86) | 24.55(0.80) | 29.17(0.75) | **< 0.0001** | **< 0.0001** | **< 0.0001** | 0.223 | **< 0.0001** |
| **Waist, cm** | 91.62(0.31) | 88.92(0.27) | 104.21(0.71) | 102.13(1.99) | 85.60(2.33) | 101.97(1.85) | **< 0.0001** | **< 0.0001** | **< 0.0001** | 0.167 | **< 0.0001** |
| **WHR** | 0.91(0.00) | 0.89(0.00) | 0.97(0.00) | 0.98(0.02) | 0.88(0.02) | 0.99(0.02) | **< 0.0001** | **< 0.0001** | **< 0.0001** | 0.342 | **< 0.0001** |

Abbreviations: MASLD = metabolic dysfunctional associated fatty liver disease, NAFLD = nonalcoholic fatty liver disease, MASLD+/NAFLD+ = individuals who met the definitions of MASLD and NAFLD, MASLD+/NAFLD- = those with MASLD but not NAFLD, MASLD-/NAFLD+ = those with NAFLD but not MASLD, MAFLD-/NAFLD- = those with not MASLD or NAFLD, TC = total cholesterol, TG = triglyceride, HDL-C = High-density lipoprotein cholesterol, CRP = C-reactive protein, UA = uric acid, BUN = blood urea nitrogen, TBiL = total bilirubin, Scr = blood creatinine, AST = aspartate amino-transferase, ALT = alanine aminotransferase, ALB = albumin, HbA1c = glycosylated hemoglobin, BG = blood glucose, C-P = C-Peptide, INS = insulin, SBP = systolic blood pressure, DBP = diastolic blood pressure, BMI = body mass index, WHR = waist-to-hip ratio, T2DM = Type 2 diabetes mellitus, FIB-4 = Fibrosis-4.
