# Supplementary material for: Comparative mortality outcomes in metabolic dysfunction-associated steatotic liver disease and nonalcoholic fatty liver disease subtypes in the United States
Source: PLoS One. 2025 Oct 31;20(10):e0335230. doi: 10.1371/journal.pone.0335230 (PMC12578175; doi:10.1371/journal.pone.0335230)
Supplement: S4 Table — Comparison of mortality between MASLD and NAFLD cohorts, further adjusted for physical activity and poverty-income ratio. (DOCX) [file pone.0335230.s004.docx]

**S4 Table** Comparison of mortality between MASLD and NAFLD cohorts, further adjusted for physical activity and poverty-income ratio.

| **Mortality** | **No. of Cases** | **Unadjusted** | | **Adjusted✝** | |
| --- | --- | --- | --- | --- | --- |
|  |  | **HR (95% CI)** | **P-value** | **HR (95% CI)** | **P-value** |
| ALL-cause |  |  |  |  |  |
| No-SLD | 2277 | ref |  | ref |  |
| MASLD+/NAFLD+ | 809 | 1.79(1.58,2.02) | **<0.001** | 1.19(1.05,1.35) | **0.02** |
| MASLD+/NAFLD- | 43 | 2.83(1.52,5.28) | **0.0013** | 0.86(0.49,1.50) | 0.6 |
| MASLD-/NAFLD+ | 31 | 0.65(0.36,1.17) | 0.15 | 0.82(0.48,1.40) | 0.6 |
| MASLD-/NAFLD- | 69 | 3.86(2.65,5.61) | **<0.001** | 2.61(1.71,3.98) | **0.004** |
| CVD-related |  |  |  |  |  |
| No-SLD | 628 | ref |  | ref |  |
| MASLD+/NAFLD+ | 226 | 2.11(1.67, 2.66) | <0.001 | 0.93(0.73, 1.19) | 0.88 |
| MASLD+/NAFLD- | 14 | 4.25(1.49,12.14) | 0.013 | 1.03(0.27, 3.91) | 0.96 |
| MASLD-/NAFLD+ | 10 | 0.81(0.31, 2.08) | 0.66 | 1.23(0.48, 3.16) | 0.88 |
| MASLD-/NAFLD- | 14 | 3.96(1.90, 8.27) | 0.002 | 2.31(0.92, 5.76) | 0.28 |
| Cancer-related |  |  |  |  |  |
| No-SLD | 571 | ref |  | ref |  |
| MASLD+/NAFLD+ | 183 | 1.58(1.19,2.09) | **0.002** | 1.26(0.99, 1.61) | 0.12 |
| MASLD+/NAFLD- | 9 | 1.87(0.64,5.42) | 0.29 | 1.03(0.28, 3.74) | 0.96 |
| MASLD-/NAFLD+ | 6 | 0.61(0.24,1.53) | 0.29 | 0.76(0.33, 1.75) | 0.69 |
| MASLD-/NAFLD- | 19 | 5.18(2.75,9.79) | **<0.001** | 4.20(1.89, 9.33) | **0.004** |

Abbreviations: MASLD = metabolic dysfunctional associated fatty liver disease, NAFLD = nonalcoholic fatty liver disease, CVD = cardiovascular disease,

HR= hazard ratio. All P were FDR-adjusted.

**✝** Model was adjusted for age, sex, race, smoking, T2DM, Hypertension, obesity, tbil, alb, ast, alt, physical activity and poverty-income ratio (PIR).

Physical activity: The inactive group was defined as individuals reporting no leisure-time physical activity. The active group comprised those meeting recommended physical

activity levels: either ≥5 sessions per week of self-reported moderate leisure-time activity (MET 3–6) or ≥3 sessions per week of vigorous leisure-time activity (MET ≥6).

The insufficiently active group included participants not classified as inactive but failing to meet the recommended physical activity criteria.

PIR was categorized as low (≤1.30; reference) or high (>1.3).
